# Therapeutic evolution in HR+/HER2- breast cancer: from targeted therapy to endocrine therapy

**DOI:** 10.3389/fphar.2024.1340764

**Published:** 2024-01-24

**Authors:** Lu-Qi Cao, Haidong Sun, Yuhao Xie, Harsh Patel, Letao Bo, Hanli Lin, Zhe-Sheng Chen

**Affiliations:** ^1^ Institute for Biotechnology, St. John’s University, Queens, NY, United States; ^2^ Department of Pharmaceutical Sciences, College of Pharmacy and Health Sciences, St. John’s University, Queens, NY, United States; ^3^ Shenzhen Hospital (Futian) of Guangzhou University of Chinese Medicine, Shenzhen, China

**Keywords:** HR+/HER2-breast cancer, clinical trial, CDK4/6 inhibitor, PI3K inhibitor, SERM, SERD

## Abstract

Breast cancer, a complex and varied disease, has four distinct subtypes based on estrogen receptor and human epidermal growth factor receptor 2 (HER2) levels, among which a significant subtype known as HR+/HER2-breast cancer that has spurred numerous research. The prevalence of breast cancer and breast cancer-related death are the most serious threats to women’s health worldwide. Current progress in treatment strategies for HR+/HER2-breast cancer encompasses targeted therapy, endocrine therapy, genomic immunotherapy, and supplementing traditional methods like surgical resection and radiotherapy. This review article summarizes the current epidemiology of HR+/HER2-breast cancer, introduces the classification of HR+/HER2-breast cancer and the commonly used treatment methods. The mechanisms of action of various drugs, including targeted therapy drugs and endocrine hormone therapy drugs, and their potential synergistic effects are deeply discussed. In addition, clinical trials of these drugs that have been completed or are still in progress are included.

## 1 Introduction

According to the American Cancer Society website, by the end of 2023, there will be approximately 300,000 new cases of breast cancer in women in the United States, and the number of deaths caused by breast cancer will reach 43,170 (American Cancer Society, 2023). Breast cancer is a significant health threat to women globally, both presently and in the future. Tests for hormone receptors (HRs), such as estrogen receptor (ER), progesterone receptor (PgR), and human epidermal growth factor receptor 2 (HER2) are able to produce positive or negative results (Kay et al., 2021). Consequently, female breast cancer is primarily categorized into four subtypes based on receptor types, i.e., HR+/HER2-, HR-/HER2-, HR+/HER2+, HR-/HER2+ (Lopez-Tarruella et al., 2022). These subtypes exhibit distinct risk profiles and treatment strategies, influenced by factors like age, race, and extent of spread. The ideal treatment for patients varies depending on their tumor subtype, cancer stage, and health condition (Howlader et al., 2014; Kay et al., 2021). Among all the subtypes, the incidence of HR+/HER2-type accounted for 74%, which was the most common subtype (Jerzak et al., 2023).

Breast cancer, which originates in the breast area, can metastasize to other parts such as lymph nodes as tumor progresses (Zhu et al., 2022). The disease is classified into stages I through IV, based on tumor size and the extent of cancer cell dissemination (Jerzak et al., 2023; Pegram et al., 2023). Early-stage breast cancer, encompassing stages 0 to III, is managed with treatments like surgery, radiotherapy, or chemotherapy, based on the patient’s physical health, tolerance, and disease severity (Torrisi et al., 2023). These treatments are supplemented with endocrine hormone therapy and targeted therapy. For stage IV or metastatic breast cancer, the disease has spread beyond the breast to other tissues or organs via blood or tissue, thereby escalating the complexity of treatment (Zhu et al., 2022; Pegram et al., 2023; Torrisi et al., 2023). Each year, nearly 41,000 individuals succumb to metastatic breast cancer (American Cancer Society, 2023). Despite the existence of numerous therapeutic agents, metastatic HR+/HER2– breast cancer continues to be difficult to overcome. Post metastasis, the 5-year survival rate for breast cancer patients is a mere 24% (Schlam et al., 2022). Hence, the urgency for discovering new therapeutic drugs apt for clinical treatment is paramount, and research in the field of breast cancer must persist. This review article encapsulates targeted therapies (including CDK4/6 inhibitors, PI3K inhibitors, PARP inhibitors, and Proteolysis-targeting chimeras) and endocrine therapies (such as Aromatase Inhibitors, Selective estrogen receptor modulators, Selective Estrogen Receptor Degraders, Complete estrogen receptor antagonists) employed in the treatment of HR+/HER2– breast cancer. It also presents an overview of the existing preclinical data, both completed and ongoing (Table 1).

**TABLE 1:** Collections of clinical trials related to strategies to treat HR+/HER2-breast cancer.

Trial	Stage	No. of subjects	Treatment	Recruitment status	Estimated complete date	Outcomes
NCT02732119 (TRINITI-1)	I	104	RP2D1 (LEE011 300 mg + Everolimus 2.5 mg + Exemestane 25 mg)	Completed	2020-02-25	CBR 65.5%
						PFS 8.0 months
	II		RP2D2 (LEE011 200 mg + Everolimus 5 mg + Exemestane 25 mg)			CBR 59.4%
						PFS 4.7 months
NCT01958021 (MONALEESA-2)	III	668	Ribociclib + Letrozole	Completed	2023-03-16	Median OS 63.9 months (95% CI, 52.4–71.0)
NCT03155997 (monarchE)	III	5,637	Abemaciclib	Active, not recruiting	2029-05-28	IDFS
			+ Standard Adjuvant Endocrine Therapy			Abemaciclib + Endocrine 92.2%
						Endocrine 88.7%
NCT01740427 (PALOMA-2)	III	666	PD-0332991+ Letrozole	Completed	2023-11-09	PFS: Palbociclib 27.6 months Placebo 14.5 months
			Placebo + Letrozole			
NCT02437318 (SOLAR-1)	III	572	Fulvestrant + Alpelisib	Completed	2023-06-09	OS: Fulvestrant 39.3months Placebo 31.4 months
			Placebo + Alpelisib			PFS: Fulvestrant 11.0 months placebo 5.7 months
NCT01698918 (BOLERO-4)	II	202	Everolimus + Letrozole + Exemestane	Completed	2021-01-13	PFS: 6 months 83.6%
						12 months 71.4%
NCT05501886 (VIKTORIA-1)	III	701	Gedatolisib + Fulvestrant	Recruiting	2026-09-30	—
			Palbociclib			
NCT02000622 (OlympiAD)	III	302	Olaparib	Active, not recruiting	2024-12-31	OS
			Physician’s choice chemotherapy			Olaparib 9.3 months
						TPC 17.1 months
NCT01945775 (EMBRACA)	III	431	Talazoparib	Completed	2021-03-05	PFS talazoparib 8.6 months
			Physician’s-Choice			TPC 5.6 months
NCT05654623 (VERITAC-2)	III	560	ARV-471	Recruiting	2028-05-15	38% clinical benefit rate at 24 weeks
			Fulvestrant			
NCT03901339	III	543	Sacituzumab Govitecan-hziy	Completed	2023-10-20	Median survival: 14.4 months [95% CI 13.0-15.7]
			Eribulin			ORR:57 [21%] patients
			Capecitabine			
NCT05104866	III	733	Dato-DXd	Active, not recruiting	2025-08-15	Reduced the risk of disease progression or death by 37 percent
			Capecitabine			Median PFS: 2 months longer, 6.9 months
			Gemcitabine			
NCT03778931 (EMERALD)	III	466	Elacestrant	Active, not recruiting	2024-08	median PFS: elacestrant 3.8 months
			Standard of Care			Standard care 1.9 months
NCT03616587 (SERENA-1)	I	403	AZD9833 palbociclib	Recruiting	2024-09-12	PFS: 75 mg AZD9833 6.3 months
						150 mg AZD9833 9.2 months
NCT05774951 (CAMBRIA-1)	III	4,300	Camizestrant	Recruiting	2027-04-19	—
			Tamoxifen			
			Anastrozole			
NCT04436744 (coopERA BC)	II	221	Giredestrant	Completed	2021-11-24	The antiproliferative activity of giredestrant is superior to that of an AI.
			Anastrozole			
			Palbociclib			
NCT04505826	I	153	OP-1250	Active, not recruiting	2024-07	well tolerated
	II					high drug exposure
						strong antitumor activity
NCT04906395 (OVELIA)	III	250	TOL2506	Recruiting	2025-04-30	—
			Tamoxifen			
			Letrozole Tablets			

## 2 Current clinical therapeutics

**FIGURE 1:**
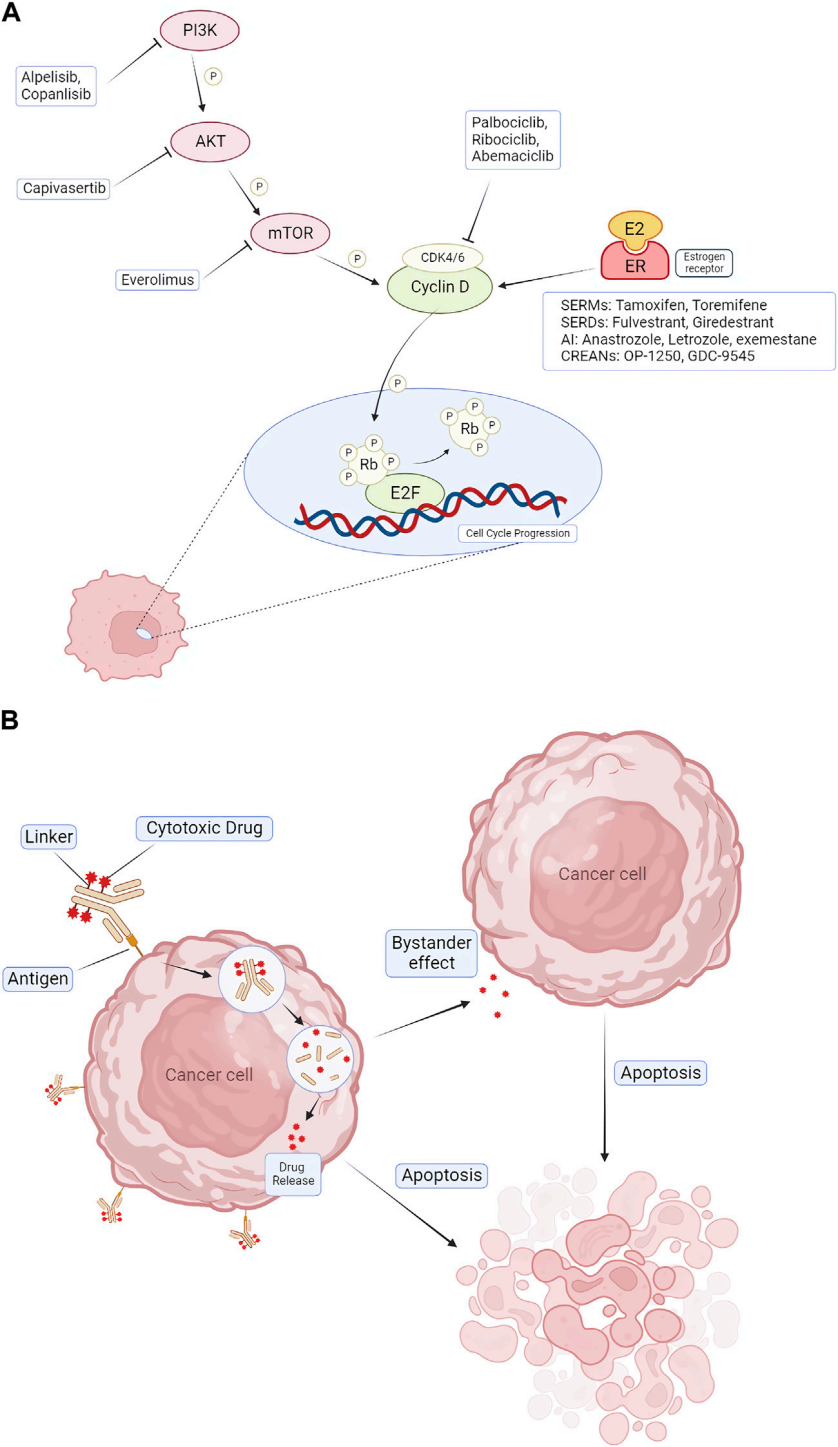
Pharmacologic mechanisms of endocrine therapy and targeted therapy for treating HR+/HER2- BC. **(A)** CDK4/6 inhibitors (palbociclib, ribociclib, and abemaciclib) block CDK4/6 activation, causing cell cycle arrest in G1 phase. Combination therapeutic strategies focus on blocking upstream of cell cycle protein D-CDK4/6 signaling, including blockade of ER via AIs, SERDs, SERMs, and blockade of the PI3K/AKT/mTOR pathway via alpelisib, capivasertib, everolimus. **(B)** The mechanism of action of ADCs. ADCs consist of three distinct components: antibodies, linkers, and cytotoxic drugs. The antibody binds to the antigen and enters the cancer cell via endocytosis, and the drug is released inside the cancer cell. It also produces a bystander effect, leading to apoptosis of nearby cells. CDK = cyclin-dependent kinase; E2F = E2 transcription factor; ER = estrogen receptor; P = phosphorylation; Rb = retinoblastoma; PI3K = phosphatidylinositol 3-kinase; mTOR = mammalian target of rapamycin; AKT = protein kinase B; AI = aromatase inhibitor; SERD = selective estrogen receptor degrader; SERM = selective estrogen receptor modulator; ADC = Antibody–Drug Conjugates.

Targeted therapy, endocrine therapy, and chemotherapy are all viable options for preliminary trials. For the systemic management of non-metastatic HR+/HER2– breast cancer, drugs like tamoxifen, a SERM, can be administered, often as a postoperative treatment (Corti et al., 2023; Lüftner, 2023). Tamoxifen, within the breast tissue, can inhibit estrogen from interacting with cancer cells, thereby preventing the cells from receiving proliferation signals, thus exerting an anti-cancer effect (Plowman, 1993; Shagufta and Ahmad, 2018). Both SERM and SERD drugs obstruct estrogen, a hormone instrumental for the growth of HR + breast cancer (Miranda et al., 2022; Lüftner, 2023). Certain SERMs also serve to reduce the incidence of breast cancer in individuals with an elevated risk of developing the disease (Ballinger et al., 2018). Additionally, these drugs can prevent and manage osteoporosis (Ballinger et al., 2018; Miranda et al., 2022). Aromatase inhibitors (AI) treat some breast cancers that depend on estrogen levels by blocking the enzyme aromatase, which converts androgens into estrogen in the body (Mamounas et al., 2019). Therefore, tamoxifen and aromatase inhibitors can only treat HR + breast cancer. In the systemic treatment of metastatic or advanced HR+/HER2– breast cancer, CDK4/6 inhibitors such as palbociclib, abemaciclib, or ribociclib are commonly used. In addition, drugs used for targeted therapy include platinum drugs, PARP inhibitors, PIK3CA inhibitors and mTOR inhibitors (Braal et al., 2021; Fontanella et al., 2022) (Figure 1).

## 3 Targeted therapy

### 3.1 CDK4/6 inhibitors

Cyclin-dependent kinases 4 and 6 play a crucial role in cell division. Their inhibitors are designed to target CDK4 and CDK6 enzymes, thereby inhibiting the progression of the cell cycle from the G1 phase to the S phase, where DNA replication takes place (Spring et al., 2019; 2020). As a result, these inhibitors can obstruct the protein synthesis of cyclin-dependent kinases, effectively blocking the proliferation of cancer cells (Piezzo et al., 2020; Zhu et al., 2022). Palbociclib (Ibrance), ribociclib (Kisqali), and abemaciclib (Verzenio) are three FDA-approved CDK4/6 inhibitors for the treatment of advanced HR+/HER2– breast cancer (George et al., 2021). These agents are typically used along with endocrine therapy drugs, such as aromatase inhibitors or fulvestrant, to suppress cancer cell growth following endocrine therapy. These drugs are primarily used for metastatic or HR+/HER2– breast cancer with a high recurrence risk (Cetin et al., 2022). Both palbociclib and ribociclib cause severe neutropenia and lung problems (Hassan and Ates-Alagoz, 2023). Therefore, abemaciclib, which can effectively reduce the risk of breast cancer recurrence after surgery, can be used for patients who cannot regularly test their blood levels (George et al., 2021; Hassan and Ates-Alagoz, 2023). Adverse effects include anemia, thrombosis and abdominal pain. In some cases, liver problems and lung inflammation may occur (Spring et al., 2019; 2020; Piezzo et al., 2020). These side effects can be managed with dose modification, supportive care, or switching to other CDK4/6 inhibitors.

Preclinical data from the TRINITI-1 (NCT02732119) (Study Details Study of Ribociclib With Everolimus + Exemestane in HR+ HER2- Locally Advanced/Metastatic Breast Cancer Post Progression on CDK 4/6 Inhibitor. ClinicalTrials.gov, 2023) clinical trial indicates that ribociclib combined with everolimus and exemestane is safe and effective for locally advanced or metastatic breast cancer. This trial involved a total of 104 patients, with 25 participating in phase I and 79 in phase II. Following the phase I trial, the dosage levels for the two patient groups RP2D1 and RP2D2 did not exceed the maximum tolerated dose (ribociclib 300 mg + everolimus 2.5 mg + exemestane 25 mg; ribociclib 200 mg + everolimus 5 mg + exemestane 25 mg; respectively) (Bardia et al., 2021). At the 24-week mark, the clinical benefit rate for RP2D1, which includes a complete response (CR), a partial response (PR), or stable disease for 6 months or longer (SD), was 65.2% (95% CI). Besides, the clinical benefit rate for RP2D2 was 59.4% (95%CI) (Bardia et al., 2019). Consequently, the results demonstrated that the combination use of ribociclib plus everolimus plus exemestane offers significant safety and clinical advantages for patients with locally advanced or metastatic breast cancer (Study Details Study of Ribociclib With Everolimus + Exemestane in HR+ HER2- Locally Advanced/Metastatic Breast Cancer Post Progression on CDK 4/6 Inhibitor. ClinicalTrials.gov, 2023; Bardia et al., 2019; 2021).

MONALEESA-2 (NCT01958021) (Study Details Study of Efficacy and Safety of LEE011 in Postmenopausal Women With Advanced Breast Cancer. MONALEESA-2 ClinicalTrials.gov, 2023) is a phase III preclinical trial that evaluated the efficacy of ribociclib and letrozole combination therapy in postmenopausal women diagnosed with HR+/HER2– advanced or metastatic breast cancer. The results revealed a notable overall survival (OS) advantage in the ribociclib group compared to the placebo group. Specifically, the median OS in the ribociclib group was 63.9 months (95% CI), while the placebo group had a median OS of 51.4 months (95% CI) (Hortobagyi et al., 2022). The median OS in the ribociclib group exceeded that of the placebo group by over 12 months (Janni et al., 2017; Hortobagyi et al., 2022). Hence, the data demonstrated that ribociclib significantly enhances both progression-free and overall survival in patients with HR+/HER2– breast cancer (Hortobagyi et al., 2022).

An open-labeled phase III clinical trial (monarchE, NCT03155997) (Study Details Endocrine Therapy With or Without Abemaciclib LY2835219 Following Surgery in Participants With Breast Cancer ClinicalTrials.gov, 2023) assessed the effectiveness and safety of abemaciclib in patients with high-risk, node-positive HR+/HER2– breast cancer. The results revealed that abemaciclib, the first CDK4/6 inhibitor used in combination with endocrine therapy, can significantly enhance the invasive disease-free survival (IDFS) in patients with node-positive HR+/HER2-breast cancer who are at a high risk of early recurrence (Johnston et al., 2023). When compared to patients who underwent endocrine therapy alone, patients treated with a combination of abemaciclib and endocrine therapy exhibited a higher IDFS rate (92.2% *versus* 88.7%, respectively; *p* = 0.01, 95%CI) (Johnston et al., 2020; 2023). This benefit was sustained post-treatment and lasted up to 4 years. These results underscore the significance of abemaciclib in treating patients with high-risk HR+/HER– early breast cancer (Hamilton et al., 2023). This clinical trial is set to continue until 2023 to further ascertain if the combination of abemaciclib and endocrine therapy can effectively enhance overall survival in these patients (Johnston et al., 2020; 2023; Hamilton et al., 2023).

Furthermore, the randomized, double-blind, phase III clinical trial (PALOMA-2, NCT01740427) (Study Details A Study of Palbociclib PD-0332991 + Letrozole vs Letrozole For 1st Line Treatment Of Postmenopausal Women With ER+/HER2- Advanced Breast Cancer PALOMA-2 ClinicalTrials.gov, 2023), building on a Phase II clinical trial PALOMA-1, compared palbociclib with letrozole in postmenopausal women with HR+/HER2– advanced or metastatic breast cancer. The results showed that palbociclib significantly extended progression-free survival (PFS) compared to letrozole (27.6 months *versus* 14.5 months, respectively; 95% CI), thereby meeting the primary endpoint of improved PFS (Im et al., 2019). However, as OS data are still immature, the secondary endpoint of OS has yet to be achieved in this clinical trial (Im et al., 2019).

### 3.2 Inhibitors of PI3K/AKT/mTOR pathway

The PI3K/AKT/mTOR pathway, an internal signaling pathway, plays a significant role in controlling cell growth, metabolism, and angiogenesis (Miricescu et al., 2020). This pathway is associated with the onset and progression of various cancers, including HR+ and HER2-breast cancer. Abnormalities such as mutations or overexpression of components within this pathway can lead to its dysregulation, contributing to different types of cancer, including breast cancer (Miricescu et al., 2020; Zhu et al., 2022). When the PI3K/AKT/mTOR pathway becomes hyperactive, it can result in unregulated cell growth and survival, thereby promoting tumorigenesis and migration (Miricescu et al., 2020; Nunnery and Mayer, 2020). This type of breast cancer is typically treated with endocrine therapies like aromatase inhibitors or fulvestrant. However, some patients may develop drug resistance to these treatments. To overcome drug resistance and enhance treatment effectiveness, drugs that target any components in the PI3K/AKT/mTOR pathway can be used in conjunction with endocrine therapy (Nunnery and Mayer, 2020; Li et al., 2021; Zhu et al., 2022). Various drugs have been developed to target this pathway, including PI3K inhibitors, AKT inhibitors, and mTOR inhibitors (Tewari et al., 2022). PI3K inhibitors, for instance, block the activity of PI3K and interfere with the pathway to slow down or prevent the mutation and proliferation of cancer cells, making them a significant therapeutic target. Currently, the FDA has approved certain drugs for the treatment of HR+/HER2-breast cancer, including the PI3K inhibitor alpelisib (Piqray) and the mTOR inhibitor everolimus (Afinitor) (du Rusquec et al., 2020; Alves and Ditzel, 2023). Other drugs, such as the AKT inhibitor capivasertib and the PI3K inhibitor copanlisib, are still under clinical trials (Alves and Ditzel, 2023).

The SOLAR-1 clinical trial (NCT02437318) (Study Details Study Assessing the Efficacy and Safety of Alpelisib Plus Fulvestrant in Men and Postmenopausal Women With Advanced Breast Cancer Which Progressed on or After Aromatase Inhibitor Treatment. ClinicalTrials.gov, 2023) conducted a comparative study of the PI3K inhibitor alpelisib plus fulvestrant and placebo plus fulvestrant in men and postmenopausal women diagnosed with PIK3CA-mutated HR+/HER2-advanced breast cancer. The results showed that compared with the addition of placebo, the median overall survival increased by 7.9 months (from 31.4 to 39.3 months) when alpelisib was added together with fulvestrant (André et al., 2021). Furthermore, the cohort treated with alpelisib plus fulvestrant produced a higher progression-free survival compared to the placebo cohort (11.0 months *versus* 5.7 months, respectively) (André et al., 2021; Ciruelos et al., 2021).

A phase III clinical trial BOLERO-2 (Study Details Everolimus in Combination With Exemestane in the Treatment of Postmenopausal Women With Estrogen Receptor Positive Locally Advanced or Metastatic Breast Cancer Who Are Refractory to Letrozole or Anastrozole ClinicalTrials.gov, 2023) assessed the effectiveness and survival rate of the mTORC1 inhibitor everolimus and the aromatase inhibitor exemestane in treating HR+/HER2-advanced breast cancer, leading to the approval of this combination. Building on these results, the BOLERO-4 clinical trial (NCT01698918) (Study Details Open-label, Phase II, Study of Everolimus Plus Letrozole in Postmenopausal Women With ER+, HER2- Metastatic or Locally Advanced Breast Cancer ClinicalTrials.gov, 2023) examined the efficacy and safety of first line everolimus and letrozole in postmenopausal patients with ER+/HER2-metastatic or locally advanced breast cancer. The outcomes confirmed that adding everolimus to aromatase inhibitors was an effective treatment strategy, with the estimated PFS rate reaching as high as 83.6% and 71.4% at 6- and 12-month spot, respectively (95%CI) (Royce et al., 2016; 2018).

Gedatolisib is an intravenous dual PI3K/mTOR inhibitor. The VIKtorI-1 clinical trial (NCT05501886) (Study Details Gedatolisib Plus Fulvestrant With or Without Palbociclib vs. Standard-of-Care for the Treatment of Patients With Advanced or Metastatic HR+/HER2- Breast Cancer VIKTORIA-1 ClinicalTrials.gov, 2023) evaluated the efficacy of gedatolisib in the treatment of advanced inoperable or metastatic HR+/HER2-patients. This clinical trial recruited patients who had previously received a CDK4/6 inhibitor or an aromatase inhibitor and had disease progression. This trial was designed to determine whether gedatolisib with fulvestrant, with or without palbociclib, could restore sensitivity to CDK4/6 inhibitors (Hurvitz et al., 2023). The mechanism of action and pharmacokinetic properties of gedatolisib are much more complex compared to other currently approved agents targeting PI3K or mTOR alone or in combination (Turner et al., 2023). This phase III clinical trial is still ongoing, and it is anticipated that gedatolisib will be able to treat a broader patient population in the future (Study Details Gedatolisib Plus Fulvestrant With or Without Palbociclib vs. Standard-of-Care for the Treatment of Patients With Advanced or Metastatic HR+/HER2- Breast Cancer VIKTORIA-1 ClinicalTrials.gov, 2023).

### 3.3 PARP inhibitor

PARP is a cellular mechanism involving poly (ADP-ribose) polymerase (PARP) that helps to repair DNA damage and regulate gene expression (Cortesi et al., 2021). PARP is activated when DNA is damaged by a number of factors such as radiation, chemicals, or oxidative stress. The PARP pathway can be used to treat certain types of cancer, particularly those that are defective in another DNA repair mechanism, which is homologous recombination (Slade, 2020; Hong and Xu, 2022). Some kind of cancers, such as breast and ovarian cancers, have mutations in the BRCA1 or BRCA2 genes that compromise their homologous recombination ability. PARP inhibitors (PARPi) causes cell death by targeting key DNA repair proteins in cells harboring germline BRCA1/2 mutations. (Slade, 2020; Cortesi et al., 2021; Wang et al., 2022). Currently FDA-and EMA-approved PARP inhibitors include olaparib (Lynparza), rucaparib (Rubraca), niraparib (Zejula), and talazoparib (Talzenna). They are often used as monotherapy or in combination with hormonal therapy or chemotherapy agents (D’Andrea, 2018; Lee et al., 2020). PARP inhibitors improve progression-free survival and overall survival in patients with HR-deficient breast and ovarian cancer. These drugs also have a good safety profile, and side effects such as fatigue, nausea, and thrombocytopenia are controllable (Zhu et al., 2020). Talazoparib and Olaparib are both oral PARP inhibitors that interfere with the PARP enzyme, making it more difficult for cancer cells with BRCA1 or BRCA2 mutations to repair DNA damage, reducing the likelihood of cancer survival and progression (D’Andrea, 2018; Slade, 2020).

In a phase III clinical trial OlympiAD (NCT02000622) (Study Details Assessment of the Efficacy and Safety of Olaparib Monotherapy Versus Physicians Choice Chemotherapy in the Treatment of Metastatic Breast Cancer Patients With Germline BRCA1/2 Mutations. ClinicalTrials.gov, 2023), scientists compared the efficacy and safety of olaparib monotherapy with chemotherapy treatment of physician’s choice (TPC) in patients with metastatic breast cancer with a germline BRCA1/2 mutation. Olaparib significantly prolonged progression-free survival in patients with germline BRCA-metastatic and HER2-metastatic breast cancer who had received second line chemotherapy drugs. The median overall survival was 19.3 months with olaparib *versus* 17.1 months with TPC (*p* = 0.513) (Robson et al., 2019; 2023). In addition, olaparib was well tolerated with no evidence of cumulative toxicity during long-term exposure (Robson et al., 2023).

The EMBRACA clinical trial (NCT01945775) (Study Details A Study Evaluating Talazoparib BMN 673, a PARP Inhibitor, in Advanced and/or Metastatic Breast Cancer Patients With BRCA Mutation EMBRACA Study ClinicalTrials.gov, 2023) evaluated the safety and efficacy of the PARP inhibitor talazoparib (BMN 673) in patients with advanced and HER2-negative metastatic breast cancer with a BRCA1/2 mutation. The results showed that, progression-free survival was superior with talazoparib *versus* TPC cohort (5.6 *versus* 8.6 months, respectively), with a similar and manageable safety profile (Hurvitz et al., 2020; Litton et al., 2020).

### 3.4 Antibody-Drug Conjugates (ADCs)

Antibody-Drug Conjugate (ADC) is a novel emerging cancer treatment method, which has developed rapidly in breast cancer. ADCs are composed of a cytotoxic payload, a cleaver or non-cleavable linker, and a monoclonal antibody (mAb) that can cause targeted cytotoxicity to tumor cells (Abelman et al., 2022; Chen et al., 2023). ADC has the ability to efficiently deliver cytotoxic medicines to cancer cells expressing certain surface antigens because it combines monoclonal antibodies with cytotoxic payloads (Abelman et al., 2022; Mark et al., 2023). Through internal circulation, ADCs attach to tumor targets, and mAb of ADC identifies and binds certain antigens on the surface of cancer cells. The complex of ADC binding to target antigen is internalized into cancer cells through receptor-mediated endocytosis (Chen et al., 2023; Mark et al., 2023). Within cancer cells, monoclonal antibodies release cytotoxic payloads, which contain large amounts of chemotherapy drugs, such as microtubule inhibitors or topoisomerase inhibitors (Najjar et al., 2022). In addition, ADCs are effective because they cause a phenomenon known as the “bystander effect,” in which the cytotoxic payload penetrates the target tumor cell’s core and has the potential to spread to other cancer cells that do not express the particular antigen that the ADC has identified (Ferraro et al., 2021; Mark et al., 2023). There are various ADC types in the treatment of breast cancer: HER2-targeted ADC, HER3-targeted ADC, Trop-2-targeted ADC and LIV1-targeted ADC (Chen et al., 2023). Most ADCs are used for metastatic triple-negative breast cancer (TNBC) or HER2+ BC, but a small number of drugs are used for HR+/HER2- BC.

Trophoblast cell surface antigen 2 (TROP-2) is a cell surface receptor that overexpressed in approximately 80% of breast cancers (Jeong and Kim, 2022). Every subtype of breast cancer was found to express it. A monoclonal antibody that has been precisely engineered to identify and bind to the TROP-2 receptor on cancer cells is used by TROP-2 targeting ADCs. Targeting the TROP-2 receptor, sacituzumab govitecan (IMMU-132, Trodelvy) is a humanized monoclonal antibody that also includes the powerful chemotherapy drug SN-38 (Marmé, 2022). Once within cancer cells, SN-38 causes damage to DNA and cell death by blocking topoisomerase I, which is a cytotoxic action. At present, several clinical studies have shown that this therapy has a good effect for patients with metastatic triple-negative breast cancer (TNBC) or HR+/HER2-MBC. In the phase III TROPiCS-02 trial (NCT03901339) (Study Details Study of Sacituzumab Govitecan-hziy Versus Treatment of Physician’s Choice in Participants With HR+/HER2- Metastatic Breast Cancer ClinicalTrials.gov, 2023), the safety and efficacy of sacituzumab govitecan were assessed in HR+/HER2– advanced breast cancer. The study showed a significant improvement in overall survival with sacituzumab govitecan compared with chemotherapy (median survival 14.4 months [95% CI 13.0 to 15.7] vs. 11.2 months [10.1 to 12.7], ORR was also significantly improved (57 [21%] patients vs. 38 [14%] patients) (Rugo et al., 2022). Sacituzumab govitecan showed a statistically significant and clinically meaningful benefit over chemotherapy, with a manageable safety profile. These data support sacituzumab govitecan as a promising option for patients with treated endocrine resistant HR+ and HER2– metastatic breast cancer (Rugo et al., 2023a).

Datopotamab deruxtecan (Dato-DXd, DS-1062a) is an ADC that also targets TROP2 and consists of a humanized anti-Trop2 IgG1 mAb. Ligation to topoisomerase I inhibitor payload deruxtecan via a stable cleavable linker. In a phase III study TROPION-Breast01 (NCT05104866) (Study Details A Phase-3, Open-Label, Randomized Study of Dato-DXd Versus Investigator’s Choice of Chemotherapy ICC in Participants With Inoperable or Metastatic HR-Positive, HER2-Negative Breast Cancer Who Have Been Treated With One or Two Prior Lines of Systemic Chemotherapy TROPION-Breast01 ClinicalTrials.gov, 2023), the efficacy and safety of Dato-DXd are evaluated in patients with inoperable or metastatic HR+/HER2– breast cancer who have received one to two cycles of systemic chemotherapy. The results showed that Dato-DXd reduced the risk of disease progression or death by 37% compared with chemotherapy ([HR] 0.63; 95% CI 0.52–0.76), provided a median PFS benefit of 2 months (6.9 months vs. 4.9 months), and was well tolerated in the context of endocrine therapy (Bardia et al., 2023).

### 3.5 Proteolysis-targeting chimeras (PROTACs)

Proteolysis-targeting chimeras (PROTACs) are multifunctional molecules consisting of two protein-binding domains, in which one bound to the E3 ubiquitin ligase and the other bound to the target protein to be degraded (Qi et al., 2021). It can induce degradation mediated by the ubiquitin-proteasome system, which is a new research direction for the development of new small-molecule drugs. PROTACs targeting ER degradation have the potential to overcome drug resistance mechanisms that limit the efficacy of traditional endocrine therapies (Saatci et al., 2021). Vepdegestrant (ARV-471), a PROTAC protein degrader targeting estrogen receptor degradation, is used to treat patients with locally advanced or metastatic ER+/HER2– breast cancer (Nieto-Jiménez et al., 2022). Clinical trials are ongoing in patients with HR+/HER2– advanced breast cancer previously treated with endocrine therapy and CDK4/6 inhibitors. Preliminary results showed that vepdegestrant has good clinical activity and tolerance (Li et al., 2022; Dogheim and Amralla, 2023). However, to determine the optimal use of PROTACs and their long-term safety and efficacy in breast cancer, monitoring and tracking of toxicological effects will be required in the later stages of clinical studies and drug development of PROTACs (Qi et al., 2021; Li et al., 2022; Dogheim and Amralla, 2023).

The VERITAC-2 clinical trial (NCT05654623) (Study Details A Study to Learn About a New Medicine Called ARV-471 PF-07850327 in People Who Have Advanced Metastatic Breast Cancer. ClinicalTrials.gov, 2023) is a global, randomized, phase III study of ARV-471 *versus* fulvestrant in ER+/HER2– advanced breast cancer. ARV-471 monotherapy was well tolerated in the phase I/II study, and a 38% of clinical benefit rate was achieved at 24 weeks in 71 patients in the phase II expansion cohort (Campone et al., 2023). ARV-471 showed reliable efficacy and good tolerability, and strong degradation of ER was found in paired tumor biopsies. Phase III clinical trials are ongoing to evaluate the safety and efficacy of ARV-471 as compared with fulvestrant in patients with advanced breast cancer (Campone et al., 2023; Hurvitz, 2023).

## 4 Endocrine hormone therapy (EHT)

Breast cancer, especially the HR + subtype, is strongly associated with female hormones (estrogen and progesterone). These hormones promote the growth and spread of HR + tumors, thus hormonal modulation is the cornerstone of therapeutic strategies (Bardia and Hurvitz, 2018). According to the American Cancer Society (ACS), the main purpose of hormone therapy is to reduce the level of estrogen or inhibit its supporting effect on tumors (Ran et al., 2022). A popular mode of adjuvant therapy is sequential administration, which is, tamoxifen for an initial 2–3 years, followed by aromatase inhibitors for another 2–3 years (Corti et al., 2023). This treatment regimen is designed to optimize therapeutic efficacy and minimize side effects. Although these therapies are effective in the treatment of breast cancer, they are associated with some certain adverse events, so that a careful risk-benefit assessment for each patient is important. Common side effects include hot flashes, bone pain, and vasomotor symptoms such as nocturnal hyperhidrosis, as well as vaginal atrophy (Harbeck et al., 2023). Rare but serious side effects include osteoporosis in premenopausal women, potential endometrial cancer, and cataracts [60]. In addition to these side effects, hormonal interventions may lead to irregular menstruation in premenopausal women. It is worth noting that the efficacy of EHT may decrease over time, so it is essential to monitor and modify the dose of treatment when possible (Fernandes et al., 2018; Thanopoulou et al., 2020). As science advances, understanding these hormonal interactions and therapeutic effects is essential to improve treatment benefits for patients.

### 4.1 Aromatase inhibitors (AIs)

Aromatase inhibitor (AI) is a type of hormone therapy that reduces estrogen levels in the body by inhibiting aromatase, which converts other hormones to estrogen (Rugo et al., 2023b). Estrogen stimulates the growth of certain breast cancer cells that have estrogen receptors, known as HR + breast cancer. Aromatase inhibitors can prevent estrogen from entering these cancer cells, thereby slowing or even arresting their growth (Lux et al., 2022). Aromatase inhibitors are mainly used to treat HR + breast cancer in postmenopausal women due to lower levels of estrogen secreted by the ovaries. They may also be used in premenopausal women undergoing ovarian suppression therapy, which prevents the ovaries from producing estrogen (Merriam and Sikov, 2011). Aromatase inhibitors can be used as adjuvant therapy, that is, after surgery or other treatment, to reduce the risk of recurrence, or as palliative therapy, that is, in patients with advanced or metastatic breast cancer, to relieve symptoms and improve quality of life (Chawla et al., 2023). There are three aromatase inhibitors approved by the US Food and Drug Administration for the treatment of breast cancer: anastrozole (Arimidex), letrozole (Femara), and exemestane (Aromasin) (Barnadas et al., 2011; Merriam and Sikov, 2011).

The ATAC clinical trial (NCT00849030) (Study Details ATAC - Arimidex, Tamoxifen Alone or in Combination ClinicalTrials.gov, 2023) compared the safety and efficacy of anastrozole with tamoxifen in postmenopausal women with early-stage HR + breast cancer. The results of this clinical trial showed that disease-free survival (DFS) was significantly improved in the anastrozole group compared with the tamoxifen group (hazard ratio HR = 0.91, 95% CI; *p* = 0.04) (Mouridsen et al., 2009). In estrogen receptor-positive patients, the recurrence rate after treatment was still significantly lower with anastrozole than with tamoxifen (HR = 0.81, 95% CI; *p* = 0.03) (Mouridsen et al., 2009). In addition, fewer deaths due to recurrence were observed in anastrozole treated group (HR = 0.87, 95% CI; *p* = 0.09). Thus, anastrozole is more effective than tamoxifen in reducing the risk of recurrence and has fewer side effects (Mouridsen et al., 2009; Martin et al., 2014).

The BIG 1-98 clinical trial (NCT00004205) (Study Details Letrozole or Tamoxifen in Treating Postmenopausal Women With Breast Cancer ClinicalTrials.gov, 2023) compared the therapeutic effects of 5 years of tamoxifen or letrozole monotherapy, or 2 years of either agent followed by 3 years of sequential therapy, in postmenopausal women with early-stage HR + breast cancer. The results showed that letrozole significantly improved disease-free survival compared with tamoxifen (5-year DFS rate was 84.0% and 81.1%, respectively; HR = 0.82, 95% CI; *p* = 0.007), and increased time to distant recurrence (TDR) (HR = 0.81, 95% CI; *p* = 0.03) (Ruhstaller et al., 2019). Thus, these results indicated that adjuvant letrozole therapy significantly reduced the risks of death related to breast cancer, disease recurrence, and distant recurrence among postmenopausal women with HR + breast cancer, as compared with tamoxifen (Regan et al., 2011; Ruhstaller et al., 2019).

A phase III, randomized, double-blind, placebo-controlled clinical trial (MA.17R, NCT00754845) (Study Details Letrozole in Breast Cancer Who Have Received 5 Years of Aromatase Inhibitor Therapy ClinicalTrials.gov, 2023) evaluated the efficacy of 5-year extension use of letrozole. This clinical trial compared letrozole with placebo in postmenopausal women with early-stage HR + breast cancer who had been taking tamoxifen for 5 years. The 5-year disease-free survival rate was 95% (95% CI) in the letrozole group and 91% (95% CI) in the placebo group (Lemieux et al., 2018). The hazard ratio for disease recurrence or contralateral breast cancer in the letrozole group *versus* the placebo group was 0.66 (95% CI; *p* = 0.01) (Lemieux et al., 2018). The results illustrated how extending AI therapy to 10 years significantly improves disease-free survival (Lemieux et al., 2016; 2018).

### 4.2 Selective estrogen receptor modulators (SERMs)

SERMs is a class of drugs that act on estrogen receptors, proteins that bind to the hormone estrogen and regulate the growth of breast cancer cells (Hanker et al., 2020). SERMs can block or activate estrogen receptors in different tissues and prevent estrogen from binding to receptors on cancer cells to prevent cancer cell proliferation (Nagini, 2017; Hanker et al., 2020). SERMs also have the ability to reduce the risk of recurrence or disease progression in women at high risk for breast cancer. SERM compounds are characterized by a flat molecular structure with a solid central core region (Michaels et al., 2023). They have competitive inhibition of the ER site, inhibiting the interaction of endogenous estrogen with its receptor, and thus weaken estrogen-mediated oncogenic transcriptional activity (Burstein, 2020; Michaels et al., 2023). Commonly used SERMs include tamoxifen (Nolvadex, Soltamox), raloxifene (Evista), and toremifene (Fareston) (Nagini, 2017; Brufsky and Dickler, 2018). These drugs have been approved by the U.S. Food and Drug Administration for the treatment or prevention of breast cancer and osteoporosis. Tamoxifen is an oral pill which acts against breast cancer in part by interfering with the activity of estrogen. In 1998, based on the results of the NSABP Breast Cancer Prevention Trial (BCPT), the US FDA approved the use of tamoxifen to reduce the incidence of breast cancer in women (Chen et al., 2022). In addition, the effectiveness of tamoxifen has been shown to increase over time, that is, treatment for 5 years is more effective than treatment for 1–2 years (Chen et al., 2022; Santen et al., 2022; Huang et al., 2023). The known severe side effects of tamoxifen are uterine cancer, blood clots, stroke, and cataracts. Adverse events associated with Tamoxifen accelerated the development of other SERMs. Raloxifene, a second-generation SERM, has been shown to reduce the incidence of breast malignancies in preclinical models, and at the same time it may reduce the risk of invasive breast cancer in postmenopausal women (Santen et al., 2022; Huang et al., 2023).

The STAR clinical trial (NCT00003906) (Study Details Study of Tamoxifen and Raloxifene STAR for the Prevention of Breast Cancer in Postmenopausal Women ClinicalTrials.gov, 2023) compared tamoxifen with raloxifene for the prevention of breast cancer in postmenopausal women who were at increased risk for breast cancer. Preliminary reports in 2006 found raloxifene to be as effective as tamoxifen in the prevention of invasive breast cancer but producing less toxicity. Results from 2010 indicated that raloxifene retains 76% of the efficacy of tamoxifen in preventing invasive breast cancer with less toxicity (Wickerham et al., 2015). These results showed that both drugs reduced the risk of invasive breast cancer by approximately 50%, but raloxifene had fewer side effects. Raloxifene retains approximately 81% of the effectiveness of tamoxifen for the prevention of invasive breast cancer and continues to approach tamoxifen for the prevention of noninvasive breast cancer (Land et al., 2014; Wickerham et al., 2015). Raloxifene also maintained a better performance with respect to uterine disease, thromboembolic events, and death (Study Details Study of Tamoxifen and Raloxifene STAR for the Prevention of Breast Cancer in Postmenopausal Women ClinicalTrials.gov, 2023; Wickerham et al., 2015).

### 4.3 Selective Estrogen Receptor Degraders (SERDs)

SERDs act by selectively targeting and degrading estrogen receptor proteins (Downton et al., 2022). SERDs specifically target ER on the surface of HR + breast cancer cells. By promoting ER degradation, SERDs effectively inhibit the ability of estrogen to trigger cancer cell growth. This inhibition of estrogen signaling can slow or arrest the growth of HR + breast tumors (Lloyd et al., 2022). Fulvestrant is a typical example of SERDs. Fulvestrant was approved by the U.S. Food and Drug Administration for the treatment of breast cancer in 2002. The drug is administered by injection every 4 weeks at a dose of 250 mg (Lloyd et al., 2022; Nagy and Jeselsohn, 2023). The drug is as effective as anastrozole in treating advanced breast cancer in postmenopausal women. Because fulvestrant must be administered by injection in a physician’s office, which causes inconvenience, scientists are trying to develop newer, more accessible, and more effective SERDs. There are several oral SERD agents currently in development and under clinical trials, such as giredestrant (GDC-9545), amcenestrant (SAR 439859), camizestrant (AZD9833), elacestrant (RAD 1901), and rintodestrant (G1T48) (Patel and Bihani, 2018). On 27 January 2023, the US FDA approved elacestrant (Orserdu) for the treatment of postmenopausal women or adult men with ER+/HER2-, ESR1-mutated advanced or metastatic breast cancer (Bhatia and Thareja, 2023; Varella and Cristofanilli, 2023). Preclinical data suggested that elacestrant is more effective when combined with CDK4/6 inhibitors or everolimus. Besides, camizestrant is being studied in metastatic HR+/HER2-, ESR1-mutated breast cancer and ER+/HER2-primary breast cancer (Blackburn et al., 2018; Wang and Tang, 2022). Drug candidates were administered orally as film-coated tablets. Giredestrant competitively binds to ER and is potent against ER-sensitive or ESR1-mutated tumors. It is more effective *in vitro* than fulvestrant and achieves higher ER occupancy *in vivo* (Patel and Bihani, 2018; Wang and Tang, 2022).

The EMERALD clinical trial (NCT03778931) (Study Details Phase 3 Trial of Elacestrant vs Standard of Care for the Treatment of Patients With ER+/HER2- Advanced Breast Cancer ClinicalTrials.gov, 2023)compared elacestrant with endocrine therapy in patients with ER+/HER2-advanced or metastatic breast cancer who had disease progression after CDK4/6 inhibitor therapy. Among 228 patients with ESR1 mutations, median PFS was 3.8 months (95% CI) with elacestrant *versus* 1.9 months (95% CI) with fulvestrant or aromatase inhibitors (Bidard et al., 2022). The most common adverse effects in the elacestrant group, which were less frequent than those in the fulvestrant or aromatase inhibitor groups, were musculoskeletal pain and nausea. The results demonstrated that elacestrant, an oral selective ER degrader, produced a more significant PFS improvement and a more controllable safety profile in the overall population and ER+/HER2-advanced breast cancer patients with ESR1 mutations, compared with standard-of-care endocrine therapy (26 Subgroup Analysis of Patients With No Prior Chemotherapy in EMERALD: A Phase 3 Trial Evaluating Elacestrant, an Oral Selective Estrogen Receptor Degrader SERD, Vs. Investigator’s Choice of Endocrine Monotherapy for ER+/HER2– Advanced/Metastatic Breast Cancer mBC, 2023; Bidard et al., 2022).

The SERNA-1 clinical trial (NCT03616587) (Study Details Study of AZD9833 Alone or in Combination in Women With Advanced Breast Cancer. ClinicalTrials.gov, 2023) compared the therapeutic effects of camizestrant with physician’s choice of endocrine therapy for patients with ER+/HER2-advanced or metastatic breast cancer whose disease progressed with the use of aromatase inhibitors and CDK4/6 inhibitors. The results showed that in patients with ESR1 mutations, the median PFS was 6.3 months in those who received 75 mg of camizestrant treatment and 9.2 months in those who received 150 mg of camizestrant treatment (Oliveira et al., 2022). In contrast, the median PFS among patients who received fulvestrant was 2.2 months. Thus, patients who received 75 mg or 150 mg of camizestrant had longer median PFS than receiving fulvestrant. Camizestrant treatment produced higher response rates of 15.7% in the 75-mg group and 20.3% in the 150-mg group, as compared with 11.5% in the fulvestrant group (Oliveira et al., 2022; Patel et al., 2023). The clinical benefit rates after 24 weeks were 48.8%, 51.0%, and 39.1%, respectively. Other phase III clinical trials, SERENA-4 (NCT04711252) (Study Details A Comparative Study of AZD9833 Plus Palbociclib Versus Anastrozole Plus Palbociclib in Patients With ER-Positive HER2 Negative Breast Cancer Who Have Not Received Any Systemic Treatment for Advanced Disease ClinicalTrials.gov, 2023)and SERENA-6 (NCT04964934) (Study Details Phase III Study to Assess AZD9833+ CDK4/6 Inhibitor in HR+/HER2-MBC With Detectable ESR1m Before Progression SERENA-6 ClinicalTrials.gov, 2023), are ongoing and will further clarify whether camizestrant is more effective or less toxic than endocrine therapy. The role of this combination regimen in the treatment of patients with HR+/HER2- ABC will also be clarified. Another ongoing phase III open-label study, the CAMBRIA-1 clinical trial (NCT05774951) (Study Details A Study of Camizestrant in ER+/HER2- Early Breast Cancer After at Least 2 Years of Standard Adjuvant Endocrine Therapy ClinicalTrials.gov, 2023), is evaluating whether camizestrant improves therapeutic effects compared with standard adjuvant endocrine therapy in patients with ER+/HER2-early breast cancer who are at intermediate-to-high or high risk for disease recurrence.

Giredestrant has potential antiproliferative and antitumor activity and is well tolerated either as monotherapy or in combination with palbociclib. There are two promising clinical trials of giredestrant that are recruiting subjects. lidERA BC (NCT04961996) (Study Details A Study Evaluating the Efficacy and Safety of Adjuvant Giredestrant Compared With Physician’s Choice of Adjuvant Endocrine Monotherapy in Participants With Estrogen Receptor-Positive, HER2-Negative Early Breast Cancer lidERA Breast Cancer ClinicalTrials.gov, 2023)is a phase III, randomized, global, multicenter, open-label study evaluating adjuvant giredestrant *versus* physician’s choice endocrine therapy (PCET) in ER+/HER2-eBC. The other clinical trial, coopERA BC (NCT04436744) (Study Details A Study to Evaluate the Efficacy, Safety, and Pharmacokinetics of Giredestrant Plus Palbociclib Compared With Anastrozole Plus Palbociclib for Postmenopausal Women With Estrogen Receptor-Positive and HER2-Negative Untreated Early Breast Cancer coopERA Breast Cancer ClinicalTrials.gov, 2023)evaluated the efficacy, safety, and pharmacokinetics of giredestrant plus palbociclib in postmenopausal women patients with untreated early-stage ER+/HER2-breast cancer (which is also called coopERA breast cancer). The results showed that the giredestrant group achieved greater complete cell cycle arrest at the time of surgery. ORR was similar with aromatase inhibitors (giredestrant + palbociclib: 50%, 95% CI; AI + palbociclib: 49%, 95% CI) (CoopERA Results Show Benefits of Giredestrant in ER+/HER2– Early Breast Cancer, 2023; Fasching et al., 2022). This clinical trial is the first trial to show that the antiproliferative activity of an oral SERD (giredestrant) is superior to that of an AI in ER+/HER2-eBC. In addition, the trial is ongoing to further evaluate the clinical benefit of giredestrant (CoopERA Results Show Benefits of Giredestrant in ER+/HER2– Early Breast Cancer, 2023).

### 4.4 Complete estrogen receptor antagonists (CERANs)

CERANs can bind to ER and completely block its function, thereby inhibiting its activation effect for cancer-promoting genes (Bryant and Dere, 1998; Hodges-Gallagher et al., 2020). CERANs can also induce ER degradation, reducing its expression level in cancer cells. Therefore, CERANs can be used to treat HR+/HER2-breast cancer. In contrast to other ER-targeting agents, such as SERMs or SERDs, CERAN directly inhibits transcriptional activation function domains 2 (AF2) and recruits nuclear receptor corepressors (N-CoR) to inactivate transcriptional activation function domains 1 (AF1), thus CERANs can effectively inhibit the estrogen signaling pathway (Shastry and Hamilton, 2023). CERANs can also overcome drug resistance that some cancer cells develop over time. Several drugs target different components of the CERAN pathway, such as OP-1250, GDC-9545, AZD9833, and LSZ102 (Patel et al., 2023). These agents are currently in clinical trials for the treatment of HR+/HER2-advanced or metastatic breast cancer, either as monotherapy or in combination with other therapies such as CDK4/6 inhibitors or PI3K inhibitors (Olema Oncology Announces Complete ER Antagonist OP-1250 Continues to Demonstrate Robust Activity in Phase 1/2 Clinical Trial Olema Oncology, 2023). OP-1250 (Palazestrant) is an oral CERAN that induces the function of SERD, that is, degradation of ER and inhibition of estrogen-stimulated breast cell proliferation and receptor degradation (ER Antagonist OP-1250 Demonstrates Continued Anti-Tumor Activity in Advanced ER+ HER2– Breast Cancer, 2023; Hodges-Gallagher et al., 2020).

The OP-1250 clinical trial (NCT04505826) (Study Details A Dose Escalation/Expansion Study of Oral OP-1250 in Subjects With Advanced and/or Metastatic HR+, HER2- Breast Cancer ClinicalTrials.gov, 2023)evaluated the safety and efficacy of OP-1250 in patients with recurrent, locally advanced, or metastatic ER+/HER2-breast cancer that had progressed after previous endocrine therapy. This clinical trial is the first human study of OP-1250 to determine dose-limiting toxicity (DLT), maximum tolerated dose (MTD), and characterize safety and pharmacokinetic (PK) profiles (Olema Oncology Announces First Clinical Data on OP-1250 in Advanced ER+/HER2- Breast Cancer Olema Oncology, 2023). Preliminary antitumor activity of OP-1250 as a monotherapy agent in HR+/HER2-metastatic or locally advanced breast cancer patients was also estimated (Hamilton et al., 2022). Preliminary results of this clinical trial showed that OP-1250 was well tolerated, with high drug exposure and strong antitumor activity (Hamilton et al., 2022; Borges et al., 2023; Olema Plans Pivotal Trial for Two-Prong Breast Cancer Treatment, 2023). In addition, two ongoing clinical trials are evaluating the combination of OP-1250 and CDK4/6 inhibitors. A phase Ib clinical trial (NCT05508906) (Study Details Phase 1b Combo w/Ribociclib and Alpelisib ClinicalTrials.gov, 2023)of OP-1250 combined with either the CDK4/6 inhibitor ribociclib or the PI3K inhibitor alpelisib in patients with MBC is now determining the antitumor activity, tolerability, and safety of this combination therapy regimen. According to the results of a phase Ib/II clinical trial (OP-1250-002, NCT05266105) (Study Details A Phase 1 Study of Oral OP-1250 in Combination With Palbociclib in HR+/HER2- Breast Cancer Patients ClinicalTrials.gov, 2023), the combination of 30–120 mg of OP-1250 with 125 mg of palbociclib was safe and well tolerated.

### 4.5 Ovarian suppression (OS)

Ovarian suppression is an older treatment for breast cancer. In women before menopause, the ovaries mainly secrete estrogen. Since some breast cancer cells use estrogen for growth, ovarian suppression mainly reduces estrogen that contributes to cancer cell growth by interfering with gonad synthesis (Bui et al., 2020; Kim et al., 2020). This approach is achieved by surgical removal of the ovaries or by a gonadotropin-releasing hormone (GnRH) analogue such as leuprolide (Lupron), which ultimately results in a marked reduction in circulating estrogen concentrations. Ovarian suppression can be used as part of breast cancer treatment in premenopausal women (women who have not yet undergone menopause) to help reduce the risk of breast cancer recurrence or the development of new breast cancer (Francis, 2023). Because chemotherapy affects the ovaries, reducing the number and quality of eggs and making pregnancy more difficult, ovarian suppression therapy can also be used to preserve fertility during chemotherapy (Kim et al., 2020; Francis, 2023). The combinations of treatment options are often chosen in clinical studies. In patients who maintained their premenopausal status or experienced a resurgence of ovarian function after undergoing chemotherapy, the inclusion of a 2-year regimen of OS along with tamoxifen demonstrated a notable enhancement in DFS when contrasted with the use of TAM alone (Baek et al., 2023).

The OVELIA clinical trial (NCT04906395) (Study Details Ovarian Suppression Evaluating Subcutaneous Leuprolide Acetate in Breast Cancer ClinicalTrials.gov, 2023), the acronym for Ovarian Suppression Evaluating Subcutaneous Leuprolide Acetate in Breast Cancer, is actively enrolling with an anticipated cohort of 250 patients. This study aims to elucidate the efficacy of TOL2506, a formulation of Leuprolide, in attenuating ovarian endocrine functionality in premenopausal women diagnosed with HR+, HER2-negative mammary carcinoma. Concurrently, the pharmacodynamic safety profile of TOL2506 in male patients with analogous breast carcinoma phenotypes will be scrutinized (Tooker et al., 2022). The investigational procedure entails a bifurcated screening regimen: an initial evaluation for ascertaining premenopausal endocrine status preceding any neoadjuvant or adjuvant chemotherapeutic intervention, followed by a comprehensive diagnostic assay post-chemotherapy or for those circumventing such therapeutic modalities. Subsequent to this vetting, participants transition into a 48-week therapeutic epoch (Study Details Ovarian Suppression Evaluating Subcutaneous Leuprolide Acetate in Breast Cancer ClinicalTrials.gov, 2023; Tooker et al., 2022).

## 5 Discussion and future prospective

The cornerstone of adjuvant therapy for HR+/HER2-BC has traditionally been endocrine therapy, which can significantly lower the risk of mortality, distant recurrence, and local recurrence (Lopez-Tarruella et al., 2022). HR+/HER2-BC patients still have the possibility of recurrence even after 5 years of adjuvant chemotherapy endocrine therapy. Therefore, a large number of clinical trials are currently conducted around advanced or metastatic BC. ADC, a targeted approach to therapy, aims to maximize efficacy while reducing systemic toxicity associated with conventional chemotherapy. The great potential of these drugs has led to a rapid increase in the number of ADCs developed and approved for cancer treatment over the past decade. Sacituzumab govitecan showed a statistically significant and clinically meaningful benefit over chemotherapy in the phase 3 TROPiCS-02 trial (Rugo et al., 2023a). On the basis of these data, sacituzumab govitecan was approved in the United States in February 2023 and in the European Union in July 2023. It is used to treat patients with unresectable, locally advanced or metastatic HR+/HER2– breast cancer in a metastatic setting who have previously received endocrine therapy and at least two additional systemic therapies (FDA, 2023). In order to overcome drug resistance brought on by endocrine therapy, PROTACs are anticipated to be employed as a novel therapeutic approach in addition to targeted therapy in the future. These agents have demonstrated benefits in terms of effectiveness, selectivity, and overcoming drug resistance in the treatment of cancer. Still, very few recent studies and data have been specifically related to breast cancer. In clinical use, PROTAC toxicity and side effects provide additional challenges. The creation and testing of CERAN and SERD are the main topics of current research. Elacestrant (Orserdu) was just officially approved for the treatment of ESR1 gene mutation and spread HR+/HER2-breast cancer on 27 January 2023 (FDA, 2023). The SERD drugs of the same class, camizestrant and giredestrant, both showed a trend to outperform the available drugs, so the clinical trials, CHERA-6 and CAMBRIA-1, were expected to achieve better results. Many preclinical studies are now at the long trial stage after recruitment was completed, while some recently identified promising pharmaceuticals are still being pushed in preclinical trials that are still in the early stages of recruitment. Numerous therapeutic approaches have produced noteworthy outcomes in phase I or phase II clinical trials, and they even offer an abundance of useful reference data for phase III and follow-up investigations.

However, the commonly used treatment options have more or less limitations, such as low bioavailability of oral drugs, tumor regrowth, harmful effects on non-targeted cells, and drug side effects caused by different metabolic states of drugs in different patients, such as fatigue, headache, musculoskeletal symptoms, thrombosis, infertility, etc (Ashrafizadeh et al., 2023). To varied degrees, these constraints restrict the therapeutic efficacy on HR+/HER2– breast cancer. Therefore, exploring new or alternative treatment options, improving drug delivery routes and drug reuse are potential research directions for HR+/HER2-breast cancer in the future. Compared to conventional therapies, nanotechnology has emerged as an innovative and potentially complementary treatment approach with improvements in medicine. Drug targeting of tumors using various nanocarriers is the main approach of nanomedicine for cancer treatment and, therefore, has excellent tumor targeting (Ansari et al., 2023). Novel drug delivery systems including niosomes, liposomes, nanospheres, and phytosomes are the preferred carrier molecules for efficient drug administration because they offer long-term drug delivery protection (Ashrafizadeh et al., 2023). Moreover, chemotherapeutic medications and natural items may be delivered via nanocapsules, which helps to overcome drug resistance in breast cancer and drastically lower the effective dosage. In addition, biomarkers for breast cancer diagnosis and detection that may be employed for early breast cancer screening and detection are also provided by nanoparticles (Malik et al., 2023). The development of new drugs for breast cancer is a long process, so drug repurposing is another strategy. Drug repurposing (DR) refers to the use of some approved drugs on the market to treat other types of diseases (Malik et al., 2022). DR can save a lot of research money, and since the safety of these drugs has been determined, the time cost is also greatly reduced. Examples of drugs now being repurposed for breast cancer include Cyclophosphamide, originally used to treat autoimmune diseases, and Goserelin, originally used to treat prostate cancer (Malik et al., 2022). In DR, reasonable dose adjustment and combination therapy will be an important direction of future research.

## 6 Conclusion

Breast cancer has a high mortality rate and is the leading cause of cancer-related death in women under 45 years old (Radecka and Litwiniuk, 2016). The difficulties in the treatment of HR+/HER2-breast cancer lie in the physical differences of women before or after menopause, tumor grade, tumor spread, and the risk of recurrence after treatment. The finest therapy alternatives are a constant source of research interest. Most of the current clinical trials have preliminarily proved the maximum tolerated dose, clinical benefit rate and progression-free survival of the corresponding combination therapy. However, its safety and efficacy still need to be further tested. Preclinical trials are a marathon that needs to maximize efficacy while minimizing adverse effects. In future developments, combination therapy will be a valuable treatment option. A reduction in the probability of adverse effects may represent a therapeutic breakthrough. Further studies are needed to solve the problem of drug resistance and reduce the side effects, so as to increase the clinical benefit rate of patients. In addition to enhancing therapeutic effects and reducing adverse effects, researchers should pay more attention to the mental health of patients. Breast removal, weight loss and severe hair loss are all tormenting the patient physically and mentally. Every patient who participates in a preclinical trial is an important hero in the development of cancer treatment. The success of preclinical trials depends on the efforts of each participant. In addition, society should pay more attention to women’s health and do a good job in the prevention and screening of breast cancer.
